# McMunn’s Elixir of Opium

**Published:** 1864-04

**Authors:** Samuel R. Percy

**Affiliations:** Professor of Materia Medica New York Medical College


### MISCELLANEOUS.

*From the N. Y. Medical Independent.*

### McMUNN'S ELIXIR OF OPIUM.

*Does it possess any superiority over other preparations of Opium?*

In the *Philadelphia Medical and Surgical Reporter* for February 27th, 1864, is a communication from "G. C. S.," of which the following is a copy:

"*Editor Medical and Surgical Reporter:*

"The following recipe for making this preparation was found among the effects of the late Dr. James R. Chilton, the celebrated Chemist of the city of New York:

"1. Take five pounds of Turkey opium, cut in small pieces and dried, and put it into a large, strong, glass jar, with a wide mouth, and pour on it sulphuric ether enough to a little more than to cover it; then stop the jar tight with a glass stopple to prevent its evaporation; set it away in a cool place, and stir it with a stick, so that all the lumps may be broken. At the end of the week drain off the ether, and again pour on as much more, and repeat, stirring it every day for a week longer, when it may be drained off as before. Then stop the jar tight, and lay it down on its side, so that all the ether that accumulates near its mouth may be drained off, and repeat doing so until the opium is all dry. Then expose it to the open air for a few days.

"The sulphuric ether extracts from the opium the *narcotine*, which is its most deleterious principle, and also deprives it of its peculiar noxious odor, so that the elixir will not smell of it thereafter.

"2. Now to free the opium of the smell of the ether, and to extract its valuable medicinal properties, boil it in water, as follows: Pour into a tin boiler four gallons of pure soft water, and when hot (not boiling) put in the opium, when a great ebullition will take place, which is owing to the evaporation of the ether. Then let it boil ten or twelve minutes, occasionally stirring it so that the lumps of the ether may be all broken and dissolved. Then set it away till the next day, when it should be strained through a cloth strainer, and if there be not four gallons of the solution, pour on the leached opium boiling water enough to make that quantity when it is strained and clear.

"When in the state of watery solution, it is better to be kept in stone crocks that will hold about two or three gallons each, and in a cool place as a cellar; after standing five or six days the clear solution should be carefully dipped off into a large tin can. The skimmings and dregs should be strained, and when clear put with the other.

"3. To this four gallons of watery solution, add five and a half gallons of alcohol, and stir the mixture thoroughly; then cover the can tight so as to prevent evaporation. After standing a few days, the clear elixir may be carefully dipped off into another can, and the dregs at the bottom strained, and when clear, poured into another.

"Now, after standing undisturbed for a few weeks, it will be fit to use. It will be equivalent to laudanum, both in its strength and the size of its dose.

"It was doubtless upon receiving this knowledge of making the preparation, that Dr. Chilton was induced to give the following testimonial:

"New York, December 29, 1836.

"Dr. John B. McMunn having made known to me the process by which he prepares his 'Elixir of Opium,' and wishing me to state my opinion concerning it, I therefore say, that the process is in accordance with well known chemical laws, and that the preparation must contain all the valuable principles of opium, without those which are considered as deleterious and useless.

"J. R. Chilton, M. D., Operative Chemist, etc.
G. C. S."

A full discussion upon this secret preparation of Opium will be of very great interest to the profession, of interest not only in deciding the question of its alleged superiority over other preparations of opium, but also as to the part the profession themselves have taken in encouraging and fostering a secret remedy which, by their recommendations, has been sold to the public at an enormous profit. It is now, and for many years has been, one of the most stringent rules of the medical profession, that every new discovery in any branch of the science, should not belong to any one individual of the profession, but should be published to the widest possible extent, for the benefit of the whole profession, that they might successfully combat the diseases they are called upon to treat.

This rule is now a part of the code of Medical Ethics, adopted by the National Medical Association. With most of the profession, *no rule* of this kind has been needed, for they have honorably given to the whole profession through the world the results of their discoveries and scientific researches. So far as our memory serves, this is the only instance in this country wherein a physician has deviated from this just rule, and been sustained by the profession in so doing. The peculiar circumstances under which this remedy was first presented to the profession, had everything to do with its pecuniary success. Dr. McMunn engaged the active co-operation of Mr. Adamson, the proprietor of the well-known drug store, No. 6, Bowery, and at that time one of the best pharmaceutists in our city, and an upright, earnest man. strong in his friendship and equally strong in his prejudices. Mr. Adamson's store was the meeting-place of a large number of medical men, and the dictum that went forth from that establishment was considered law. Mr. Adamson was authority, and no man could remain his friend who dared to dispute what he authoritatively asserted; he stated that McMunn's Elixir of Opium was superior to every other preparation of that drug, and induced the profession who visited his store to try it. To any one who raised the question of *secresy*, Dr. Chilton's recommendation was shown, and it was stated that Dr. McMunn had been at great expense and trouble in the discovery of his secret, and that he was poor and absolutely needed the re-payment of his outlay.

Dr. McMunn may have gone to others before he put himself in the hands of Mr. Adamson, but my memory does not carry me back further than the time I knew them to be associated together. While at Mr. Adamson's store one day, I questioned the propriety of the profession sustaining this *secret* remedy, and stated as my belief, from experiments that I had tried, that it contained no advantages over some other preparations of opium. I was taken up pretty sharply by Mr. Adamson, who in all honesty and earnestness, differed from me on all the points raised, but I offered to back my assertions by proof. A few days afterwards Mr. Adamson and Dr. McMunn called upon me, wishing to interest me in the success of this remedy. Dr. McMunn explained to me that this preparation of opium contained all the active principles of the drug, excepting that it was deprived of much of the bad taste and smell, and was entirely robbed of its

*narcotic* property, that it was *denarcotized.* I asked him if he meant by that, that the morphia was taken from it, or that morphia was the narcotic principle? He said that morphia was not the narcotic principle, but that there was another principle in opium which was a stimulating narcotic, that caused headache and constipation, and that his preparation was deprived of that injurious principle, leaving all the good properties of the drug behind. I asked him if *narcotine* was that principle which he abstracted, and after some hesitation and circumlocution, he admitted that it was. I told him that as a chemist I had abstracted narcotine from opium many times; that I had tried its effects on others and upon myself, and that I could not discover that it possessed the slightest *narcotic* property, and that I had several times taken it in two-grain doses, without any apparent effect.

My two visitors asserted dictatorially and authoritatively, that I was a dangerous and an ignorant man; that Dr. B. and Dr. C., and all the best physicians of the city knew and acknowledged the advantages of this preparation over all and every other preparation of opium, and that my ignorance was only equalled by my insolence in presuming to think differently from the universally accepted doctrine of the profession.

After this interview they told their friends that I was crazy, and not a little dangerous. Not much liking the treatment I received, I tried many experiments to imitate this secret remedy, and after repeated trials made by exactly the same process that we have presented at the beginning of this article, a preparation that could not be distinguished from it. But nobody would use it; it could be sold at six cents an ounce, whereas the *genuine* was worth twenty-five cents; of course it was worthless. But time proves all things, and after awhile we had scientific facts coming to us from across the water—by transmission across the ocean they were entitled to consideration. Professor O'Shaughnessy and others proved to us that *narcotine* possessed no narcotic property whatever, and that it had been given in a dose of 129 grains at one time, without exciting any obvious effects. It is now known amongst students of materia medica as *anarcotina,* and as safe anti-periodic in 20-grain doses. It would seem that this acknowledged fact should have completely put an end to the use of McMunn's *denarcotized* Elixir of Opium, but charlatans are always ingenious; the theory of denarcotization was dropped, and it was claimed that the elixir was superior to all other preparations of opium, because it did not affect the brain, leave the patient with a headache, or confine the bowels. I think any person can entirely disprove all these assertions, by simply depriving the elixir of its morphia alone, and using the remaining fluid, and finding thereby its utter uselessness. The process adopted by this recipe of McMunn's leaves in the elixir the morphia, the narcine and extractive matters only, and deprives the opium of its pseudo-morphia, codeia, narcotina, thebaina, meconine, fatty matter, and resin. The narcine is quite inert in doses of two grains, which is a larger quantity than is contained in a dozen bottles of the elixir; the balance of the liquid may be taken in doses of an ounce after the morphia alone is precipitated from it. It therefore seems demonstrable that this elixir is nothing more than a solution of impure morphia.

It has been my fortune to have patients under my care, who have used this McMunn's Elixir of Opium, and they have asserted that they could take it with certain relief of their symptoms, when any other preparation of opium always affected them injuriously, or made them worse. I have repeatedly given to such parties a solution of S. Morphine, in proof spirits, with just sufficient laudanum added to give it color and smell, and while the patients have supposed they were taking the elixir, it has always acted as well.

There are many medical men who now use this elixir, and I frequently hear the assertion from them that it acts better than any other preparation of opium. This, I conceive, is owing to their being accustomed to its use, and being thereby better able to graduate the dose of this than of the stronger and weaker solutions of morphia of our drug stores.

For some years past the preparation of this Elixir of Opium has passed into the hands of a firm of enterprising wholesale druggists, who bought the recipe as a business speculation; they have advertised it largely, and it has been very remunerative to them. Of the physicians who use it in this city, I do not call to mind one who would not condemn a younger brother practitioner who should write a prescription for, or recommend to their patients any of the advertised patent medicines of the day, or any of the patented surgical appliances, and I have heard more than one of these gentlemen refuse to give any consideration in the Academy of Medicine, to pretended surgical appliances for which patents have been obtained. In this I fully sustain them, but I should think it would become them better to be consistent.

The publication of this recipe by "G. C. S.," the correspondent of the *Medical and Surgical Reporter*, deserves the thanks of the whole profession. He has mine most deeply, not only on account of putting this matter in its true light, but also because the recipe itself proves that my investigations and assertions of the last twenty-five years are correct in every particular.

SAMUEL R. PERCY, M. D.,
Professor of Materia Medica New York Medical College.

